# Effects of N-glycosylation of the human cation channel TRPA1 on agonist-sensitivity

**DOI:** 10.1042/BSR20160149

**Published:** 2016-10-06

**Authors:** Timothy J. Egan, Mario A. Acuña, Marcy Zenobi-Wong, Hanns Ulrich Zeilhofer, David Urech

**Affiliations:** * Cartilage Engineering & Regeneration Lab, the Department of Health, Science & Technology, The Swiss Federal Institute of Technology (ETH), 8049-Zurich, Switzerland; † Numab AG, 8820-Wädenswil, Switzerland; ‡ Institute of Pharmacology & Toxicology, University of Zurich (UZH), 8057-Zurich, Switzerland

**Keywords:** cinnamaldehyde, glycoprotein, glycosylation, menthol, TRP channels, TRPA1

## Abstract

Determining the functional significance of post-translational modifications advances our understanding of many broadly-expressed proteins, and particularly ion channels. The enzymes that catalyse these modifications are often expressed in a cell-type specific manner, resulting in considerable structural diversity among post-translationally modified proteins that are expressed across a variety of cell types. TRP channels exhibit notably variable behaviour between cell types *in vitro* and *in vivo*, and they are frequently modified with N-glycans that contribute to protein function. TRPA1 possesses two putative N-linked glycosylation sites at Asn^747^ and Asn^753^ that have not yet been studied in detail. In the present study, we show that both of these sites can be modified with an N-glycan and that the glycan at position Asn^747^ modulates agonist-sensitivity of TRPA1 *in vitro*. Additionally, we found that N-glycosylation also modulates cooperative effects of temperature and the agonist cinnamaldehyde (CA) on TRPA1 channel activation. Collectively, these findings suggest a dynamic role played by the N-glycosylation of human TRPA1. They also provide further evidence of the versatility of N-glycans and will assist in efforts to fully understand the complex regulation of TRPA1 activity.

## Abstract (precis)

Our experiments confirm N-glycosylation of the human cation channel TRPA1 and suggest a role of the N-glycan at position Asn^747^ in determining channel sensitivity to various agonists. Further, the activity-modulating effects of TRPA1 N-glycans are evidently influenced by temperature.

## INTRODUCTION

Transient receptor potential (TRP) channels (reviewed in [1]–[7]) are a family of nonselective, cation-permeable integral membrane proteins that commonly act as receptors in various sensory processes. Functional TRPA1 channels are composed of four subunits, each containing six transmembrane regions (S1–S6), two extracellular domains (E1 and E2) and an ion-permeable pore formed by the S5–S6 regions. TRP channels are modulated by diverse chemical and physical stimuli and simultaneous exposure to combinations of these stimuli often trigger unique channel responses, revealing cooperative effects. In addition, a single TRP channel gene can give rise to many structurally and functionally diverse channels due to the ability of subunits to heteromultimerize as well as display post-/co-translationally-added modifying groups such as N-glycans [8].

TRP channels are highly conserved, and studies have described the N-linked glycosylation of many of them [8–16]. This common protein modification entails the co-translational addition, and subsequent processing, of an oligosaccharide to side-chains of lumenal asparagine residues that are displayed within a specific N-*X-*S/T consensus sequence (where *X* is any amino acid other than proline) [17]. In general, a protein-bound N-glycan can exhibit one of three structures: high-mannose, hybrid or complex [17]. The role of N-glycans identified in studies has ranged from involvement in protein folding and cellular localization to protein function [8,18]. In the case of TRP channels, N-glycans have been shown to influence cell-surface (CS) expression [9,11,12,14,15], sensitivity to agonists [13], activity regulation [9,16] and temperature sensitivity [12].

The TRP channel ‘ankyrin’ 1 (TRPA1) is most prominently expressed on nociceptive afferent fibres of dorsal root ganglia (DRG) and trigeminal ganglia (TG) neurons and is activated by various exogenous and endogenous chemical stimuli *in vivo.* In addition, channel responses to cold (<17°C) and mechanical stimulation have been reported [19–36]. TRPA1 is structurally distinguished from other members of the TRP family by the 14–18 ankyrin repeat motifs along its cytosolic N-terminal domain [33,34,37]. Its N-terminus is further characterized by reactive cysteine and lysine residues, which act as sites for reversible covalent binding by membrane-permeable electrophiles exhibiting sulfhydryl-/amine-reactive groups [26,38]. Reversible covalent binding of these residues is the mechanism by which several pungent chemicals, such as cinnamaldehyde (CA) and allyl isothiocyanate (AITC), activate TRPA1 [26,38].

**Figure 1:**
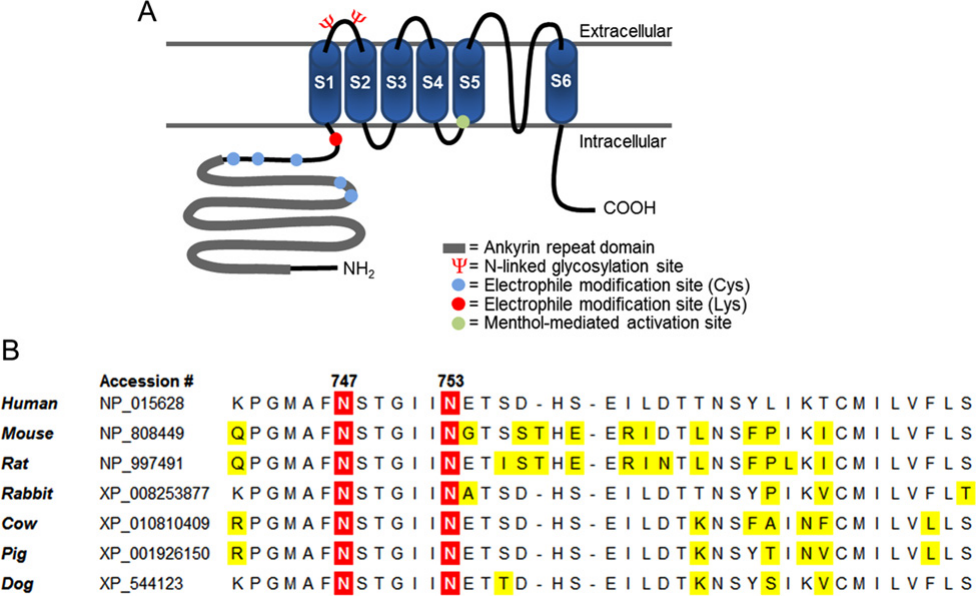
Structure of TRPA1 and orientation of its N-glycosylation site (**A**) Graphical representation of the structure of an hTRPA1 subunit, including the N-glycosylation sites along the E1 domain, the N-terminal ankyrin repeat domain, the locations of reactive cysteine (five) and lysine (one) residues, and the location of the two residues on S5 critical to menthol activation (S873 and T874). (**B**) Sequence alignment of the E1 domain of TRPA1 (retrieved from the NCBI database) across various mammalian species, revealing the conservation of both glycosylation sites. Residues highlighted in yellow indicate non-identity with human TRPA1.

Mammalian TRPA1 contains two highly conserved (Figures 1A and 1B) lumen-exposed, N-glycosylation consensus sequences (at Asn^747^ and Asn^753^) along its E1 domain. Meanwhile, Western blot (WB) analysis of denatured lysates from cells expressing human TRPA1 (hTRPA1) display a characteristic doublet band at the approximate position of the channel's subunit, presumably due to the disparate weights of ‘mature’ and ‘immature’ glycoprotein. The present study sought to confirm the presence–and characterize the role–of hTRPA1-linked glycans with the aim of offering valuable insights into protein function and regulation.

Our present data confirm the di-glycosylation of TRPA1 and indicate a functional role for the glycan at position Asn^747^. These findings present further evidence of the functional significance of N-glycosylation, especially as it relates to ion channels.

## EXPERIMENTAL

### Cloning wild-type and mutant hTRPA1-FLAG

For subsequent cloning and transfection, hTRPA1/pENTR223.1 cDNA (NCB Accession #: NP_015628) was supplied by Fisher Scientific AG. hTRPA1-FLAG cDNA was amplified and cloned into a pcDNA™5/FRT (Life Technologies) transfection vector between KpnI and XhoI restriction sites, generating the open reading frame (ORF) using the following primers: forward, 5′-AAAGGTACCATGAAGCGCAGCCTGAGG-3′; reverse, 5′-ATTCTCGAGCTATTTGTCGTCGTC GTCTTTG-TAGTCAGGCTCAAGATGGTGTGTTTTTGC-3′. To produce cDNA with Asn-Gln mutations encoded, site-directed mutagenesis was performed by overlapping PCR, using the mutating primers (N747Q: forward 5′-CCAGGAATGGCTTTCCAGT-CAACTGGCATC-3′; reverse 5′-GATGCCAGTTGACTGG-AAAGCCATTCCTGG-3′; N753Q: forward 5′-CAACTGGCA-TCATCCAGG AAACTAGTGATC-3′; reverse, 5′-GATCACTA GTTTCCTGGATGATGCCAGTTG-3′) in combination with primers for cloning between HpaI and BamHI restriction sites located within the hTRPA1 ORF (forward, 5′-ATT-GTTAACACAACCGATGGATGTCATGAGACC-3′; reverse, 5′-ATTGGATCCTGTAAATTCAGGAGGATGTAAAAGC-3′).

### Transient transfection of HEK293 cells and Western blot experiments

HEK293 cells were seeded on poly-L-lysine (0.01% solution; Sigma–Aldrich) treated growth surfaces at ∼60000 cells/cm^2^ and grown for 16–24 h in Dulbecco's Modified Eagle's Medium (Sigma–Aldrich) supplemented with 10% heat-inactivated fetal calf serum (v/v), 1% Pen-Strep (v/v) and 2 mM L-glutamine (complete DMEM). The cells were transfected using the jetPRIME™ system (Polyplus Transfection), according to manufacturer's instructions, and the culture medium was exchanged ∼12–16 h after transfection. Cells were always harvested 36–48 h after transfection.

To harvest, intact cells were rinsed in ice-cold, 1× PBS, pH 7.4, without Ca^2+^ or Mg^2+^ (PBS) and treated with lysis buffer (1% Triton X-100 in PBS w/protease inhibitors) for 30 min on ice, agitating intermittently. The samples were then centrifuged at 10500 ***g*** for 15 min at 4°C, and the pellets were discarded. The protein concentration of samples was then determined by the DC™ Protein Assay (Bio-Rad Laboratories) according to manufacturer's instructions, and sample concentrations and volumes were equalized. To denature the lysate samples, SDS/PAGE Loading Buffer [0.4M Tris/HCl (pH 8.5), 5% SDS (w/v), 40% glycerol (v/v), 0.5 mM EDTA, 0.01% Bromophenol Blue (w/v), 50 mM DTT] was added (1:2) and the samples were incubated for 30 min at RT and then boiled at 95°C for 5 min. For WB detection, M2 mouse anti-FLAG® mAb (monoclonal antibody, Sigma–Aldrich, Catalogue #: F3165) served as the primary antibody and a peroxidase-conjugated, rabbit anti-mouse IgG (Jackson ImmunoResearch, Catalogue #: 315-035-046) was used as a secondary antibody. Lectin-mediated hTRPA1 detection was also performed, applying a concavalin A (ConA)-peroxidase conjugate (Sigma–Aldrich, Catalogue #: L6397) to transfer membranes displaying immobilized, purified hTRPA1-FLAG (purification method described in next section). WB data analysis was performed using signal intensities (a.u.) normalized to total protein transferred, which was revealed by staining transfer membranes with 1× Amido Black Staining Solution (Sigma–Aldrich) and destaining overnight in 15% methanol and 10% acetic acid in ddH_2_O. To compare protein expression levels, the combined, normalized signal intensities of all TRPA1 subunit glycoforms detected in the TRPA1 mutant samples–mature and immature glycoprotein (in N747Q and N753Q samples) or non-glycosylated protein (in N747/753Q samples)–were compared with the combined signal intensities of all TRPA1 subunit glycoforms detected in wild-type (WT) samples.

### Endo H and PNGase F digestion of denatured protein

HEK293 cell-transfections were carried out in flasks with a 75 cm^2^ growth-surface area. After incubation, the cells were rinsed three times in ice-cold PBS and lysed in 1 ml lysis buffer. WT or mutant hTRPA1-FLAG was immuno-precipitated out of whole-cell lysates (CLs) by incubation with 50 μl M2 anti-FLAG® magnetic beads (Sigma-Aldrich), rotating the samples overnight at 4°C. After rinsing the beads 3× with ice-cold PBS, the proteins were eluted from the beads with 100 μl, 100 mM Glycine-HCl, pH 2.8 (rocking the samples for 15 min at RT), and the pH of the eluate was promptly adjusted by addition (1:30) of 1M Tris-HCl, pH 9.5.

From each eluate, three 9 μl samples were aliquoted and the protein samples were denatured for subsequent glycosidase (or mock) digestion, according to manufacturer's (New England Biolabs) instructions, with either 75000 units/ml Endo H, 50000 units/ml PNGase F or ddH_2_O (mock). The samples were incubated with their respective glycosidase (or ddH_2_O) for 2 h at 37°C. The samples were then analysed by Western blot.

### Cell-surface biotinylation

HEK293 cell transfections were carried out in flasks with a 25 cm^2^ growth-surface area. After incubation, the intact cells were rinsed three times in ice-cold PBS and incubated in 1.0 mg/ml EZ-Link Sulfo-NHS-LC-Biotin (Thermo Scientific) in PBS for 30 min at 4°C. The biotin was quenched by washing the cells 3× in ice-cold, 100 mM glycine–PBS, and then three additional washes with ice-cold PBS were performed. The cells were lysed in 500 μl lysis buffer. After equalizing the protein contents, aliquots from each lysate sample were set aside (CL samples) and the remaining lysate was incubated with 50 μl Pierce™ Streptavidin Agarose (Thermo Fisher Scientific), rotating overnight at 4°C.

The agarose was pelleted by centrifugation at 1000 ***g*** and 4°C, supernatants were discarded and the agarose was washed 6× total: 3× in Wash Solution A (50 mM Tris/HCl, pH 7.4, 100 mM NaCl, 5 mM EDTA), 2× in Wash Solution B (50 mM Tris/HCl, pH 7.4, 500 mM NaCl) and 1× in Wash Solution C (50 mM Tris/HCl, pH 7.4). The streptavidin-affinity precipitate was eluted from the beads with 80 μl SDS/PAGE Sample Loading Buffer, incubating for 30 min at RT (while gently agitating) and then boiling for 5 min at 95°C. All samples were then analysed by WB using signal intensities normalized to total precipitate transferred, as determined by detection with streptavidin-peroxidase. To confirm the absence of substantial intracellular contamination in the cell-surface fraction, an anti-glyceraldehyde-3-phosphate dehydrogenase (anti-GAPDH) IgG (Sigma–Aldrich, Catalogue #: G9545) was applied as a primary antibody to be detected by an HRP-conjugated goat, anti-rabbit IgG (Jackson ImmunoResearch, Catalogue #: 111-035-046).

### Membrane depolarization assays on the FlexStation 3

HEK293 cells were seeded in poly-L-lysine-treated, 96-well plates (∼65000 cells/cm^2^) and incubated for 16–24 h. Transfection samples (100 μl jetPRIME™ Transfection Buffer and 2 μl jetPRIME™ Transfection Reagent per 1 μg plasmid) were diluted in pre-warmed complete DMEM at a concentration of ∼1.6 μg plasmid/ml. The old medium was discarded, and ∼220 ng/cm^2^ of the appropriate plasmid were added to each well. The cells were then incubated for 18–26 h.

The medium was discarded and 100 μl 1× Membrane Potential Assay Kit RED Dye in Component B Buffer (Molecular Devices, Inc.), a voltage-sensing dye, were added to each well and the plates were incubated for 30 min at either 37 or 23°C. The plates were analysed in a FlexStation® 3 scanning fluorometer (Molecular Devices) at 525 nm excitation and 565 nm emission (550 nm cut-off) wavelengths. Initiation of the scanning programme resulted in the transfer of 0.02–1000 μM of the TRPA1 agonist–CA, AITC or menthol–to each well. Fluorescence intensity data were collected using SoftMax® Pro v5.4 (Molecular Devices) for 2.5 min after application of the agonist. These data were then baseline-subtracted, yielding the relative fluorescence units (RFU) presented here. Normalized fluorometric data are presented as % maximum RFU. All dose–response curves were fitted to data up to the maximal channel response using GraphPad Prism® 6 software.

### Electrophysiological recordings

HEK293 cells were maintained in complete DMEM and plated on 35 mm dishes 16–24 h before transfection. Cells were co-transfected with 1 μg of plasmid encoding hTRPA1-FLAG (WT or mutant) and 1 μg of plasmid encoding eGFP (used as a marker of successful transfection) and then incubated for 16–24 h. On the day of experiment, the medium was discarded and replaced with pre-warmed extracellular solution (37°C) containing (in mM): 150 NaCl, 10 KCl, 2.0 CaCl_2_, 1.0 MgCl_2_, 10 HEPES (pH 7.4) and 10 glucose. Whole-cell patch-clamp recordings were performed at a holding potential of −60 mV using an EPC7 amplifier and Patch Master v2.11 software (HEKA Elektronik, Lambrecht-Pfalz, Germany). Recording electrodes (3.5–4.5 MΩ) were pulled from borosilicate glass capillaries and filled with internal solution containing (in mM): 120 CsCl, 10 EGTA, 10 HEPES (pH 7.35), 4 MgCl_2_, 0.5 GTP and 2 ATP. Only eGFP-positive cells were used for recordings. Whole-cell TRPA1-mediated currents were activated by 0.5–1.5 min application of CA fed through an outlet tube (200 μm ID) of a custom-designed, gravity-fed, microperfusion system positioned 50–120 μm from the recorded cell. The temperature of solution in the cell's microenvironment was maintained at 37°C using a Heat/Cooled Temperature Control System (AutoMate Scientific®).

## RESULTS

### hTRPA1 is glycosylated *in vitro*

To investigate N-glycosylation at both Asn^747^ and Asn^753^, we applied three strategies: (1) site-directed mutagenesis of cDNA to replace the critical asparagine residues with structurally similar glutamine [10,12–16]; (2) glycosidase digestion of hTRPA1-FLAG purified from CLs [10,11,14] and (3) lectin-mediated detection of glycosylated hTRPA1-FLAG [15].

**Figure 2:**
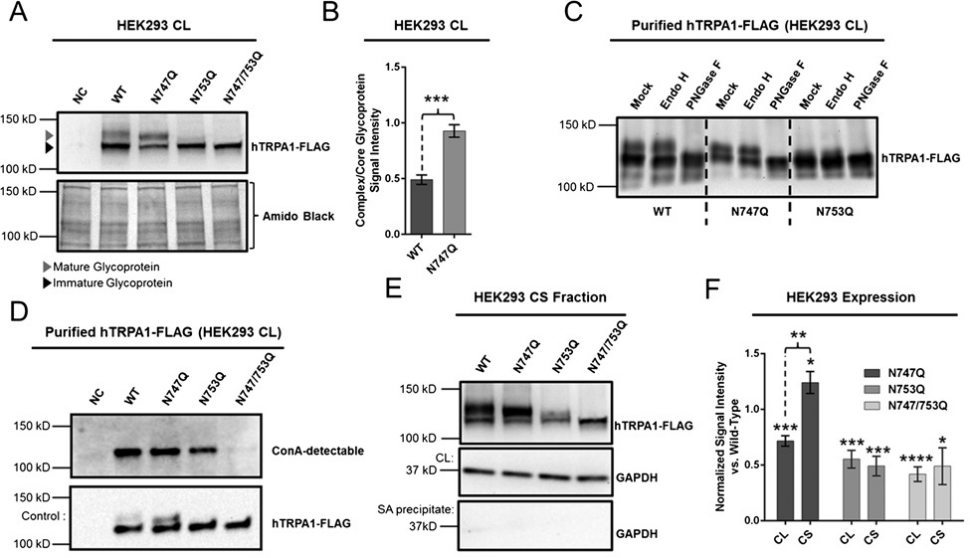
Effects of N-glycosylation on TRPA1 expression (**A**) M2-mediated detection of hTRPA1-FLAG WT and mutants harvested from CLs of transiently-transfected HEK293 cells. (**B**) Ratio of complex- to core-glycosylated hTRPA1 analysed by a Student's *t* test (*n*=5 independent transfections), ***P<0.001; data are expressed as mean ± S.E.M. (**C**) M2-mediated detection after glycosidase (PNGase F or Endo H) or mock digestion of M2-purified hTRPA1-FLAG WT, N747Q and N753Q mutants (representative of two independent experiments). (**D**) ConA-mediated detection of M2-affinity-precipitated hTRPA1-FLAG glycoforms (representative of two independent experiments). (**E**) M2-mediated detection of CS hTRPA1-FLAG isolated by biotinylation of intact cells followed by streptavidin-affinity precipitation. (**F**) Relative, normalized [to total protein content (CL) or to total streptavidin-affinity precipitate (CS)] expression of mutant compared with WT hTRPA1-FLAG analysed by a Student's *t* test (*n*=5 independent transfections), *P<0.05, **P<0.01, ***P<0.001, ****P<0.0001; data are expressed as mean ± S.E.M.

WB analysis of HEK293 cells transiently expressing hTRPA1-FLAG WT and N747Q and N753Q mutants revealed a doublet band at the estimated size of the TRPA1 subunit (∼128 kDa; Figure 2A). This doublet was conspicuously absent from lysates containing the N747/753Q double-mutant (Figure 2A). Further, the TRPA1 glycoforms exhibiting the lowest electrophoretic mobility lost considerable signal intensity when subjected to PNGase F, but not Endo H, digestion (Figure 2C), suggesting that the mature glycoprotein is modified with either a hybrid or complex glycan (i.e. one bearing terminal sialic acid) at both asparagine residues. It also seems likely that the faster-migrating TRPA1 subunits in the respective doublets have been modified with a core glycan because, unlike the N747/753Q double-mutant, they were detected by ConA (Figure 2D), which possesses an affinity for terminal α-D-mannose or α-D-glucose [39]. Moreover, ConA failed to detect any of the slower-migrating subunits in the doublets, suggesting that both N-glycosylation sites ultimately bear a complex glycan possessing three or more antennae [39].

### Expression of hTRPA1 is affected by N-Q mutation

Over the course of five independent HEK293 transfections, the presence of either single N-Q mutation reduced total protein expression as measured by normalized (to total protein content) WB signal intensities relative to WT (mean ± S.E.M.) (N747Q: 0.72±0.05; N753Q: 0.55±0.08), whereas the N747/753Q mutant consistently displayed the weakest signal intensity (0.42±0.06; Figure 2F). Unexpectedly, WB analysis also indicated that the absence of the Asn^747^ glycosylation site enhances the glycoprocessing of the N-glycan at Asn^753^, as determined by differences in the ratio of complex-/core-glycosylated protein signal intensities (N747Q: 0.93±0.07; WT: 0.49±0.04; Figure 2F).

In addition to the differences observed in overall protein expression, we detected disparate CS expression between mutant and WT hTRPA1 after five independent HEK293 transfections (Figures 2E and 2F). The lower cell-surface expression of N753Q and N747/753Q mutants relative to WT (N753Q: 0.49±0.09; N747/753Q: 0.49±0.16) was apparently consistent with the reduction in total protein expression, determined by analysis of mean ratios of WT-relative cell-surface expression to WT-relative total hTRPA1 expression (N753Q: 0.88±0.07; N747/753Q: 1.08±0.27). However, expression of hTRPA1 N747Q at the CS was evidently greater than WT (1.24±0.10), indicating that removal of the glycan at position Asn^747^ results in a greater proportion of total protein localizing to the cell membrane (1.76±0.19).

**Figure 3:**
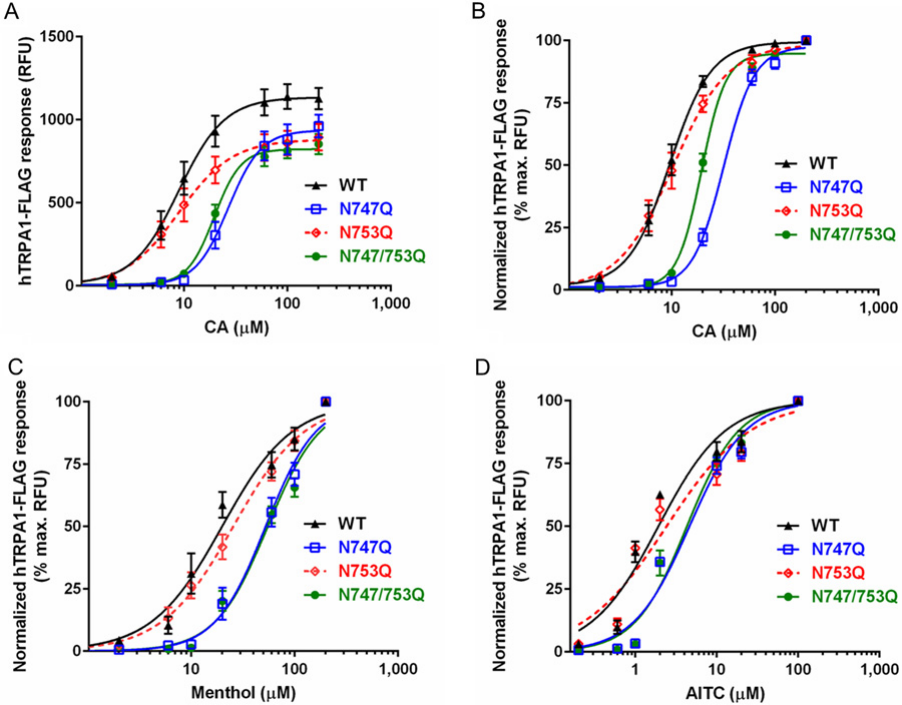
Effects of N-glycosylation on TRPA1 agonist-sensitivity Dose–response curves depicting membrane depolarization of HEK293 cells expressing hTRPA1-FLAG glycoforms as measured on a FlexStation® 3 (data are expressed as mean ± S.E.M). (**A**) Response of hTRPA1-FLAG to indicated concentrations of CA. (**B**) Normalized (% maximum RFU) response of hTRPA1-FLAG to indicated concentrations of CA. (**C**) Normalized response of hTRPA1-FLAG to indicated concentrations of menthol. (**D**) Normalized response of hTRPA1-FLAG to indicated concentrations of AITC.

**Figure 4:**
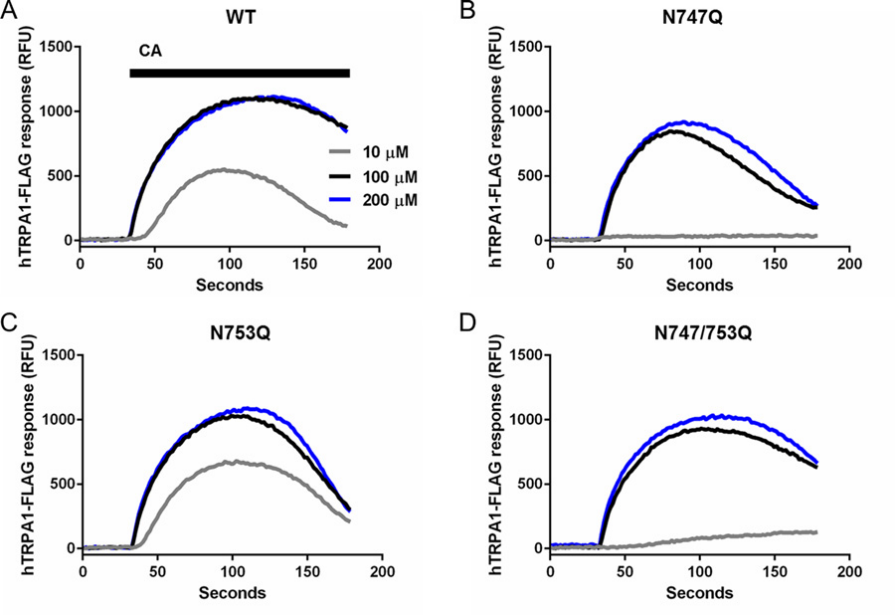
Robust activity of both N-glycosylated and non-N-glycosylated TRPA1 Fluorescence traces collected on a FlexStation® 3 (set to 37°C); representative of four independent experiments.

**Figure 5:**
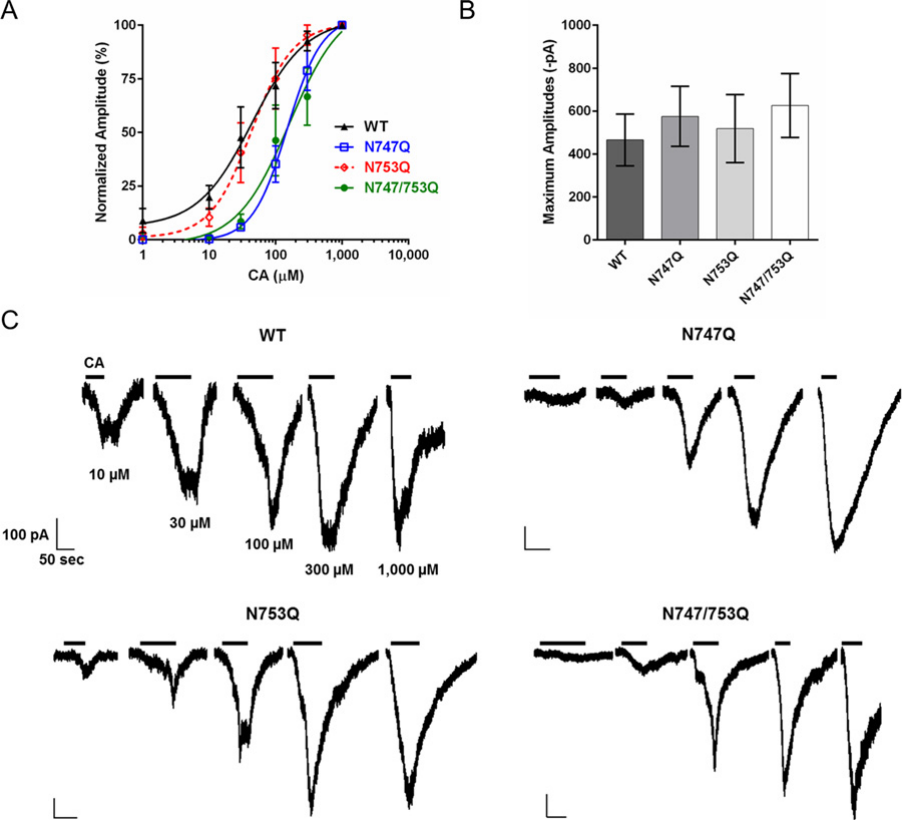
Electrophysiological assays confirm reduced sensitivity of N747Q mutants Data collected in whole-cell patch-clamp set-up (data are expressed as mean ± S.E.M). (**A**) Normalized peak amplitudes (% maximum amplitude for the respective cell) after application of indicated concentrations of CA (*n*≥4 cells each); CA was applied until current stabilized or began to desensitize during application. (**B**) Average maximum amplitudes of cells expressing each respective glycoform (*n*≥4 cells each); the amplitudes were compared by one-way ANOVA (*P*>0.05). (**C**) Examples of patch-clamp current traces for each hTRPA1 glycoform during CA application/removal.

Despite the WB results we obtained, we did not observe significant differences between TRPA1 variants in maximal changes in relative fluorescence and peak amplitudes in our fluorometric voltage measurements and electrophysiological current recordings respectively (Figures 3A, 4 and 5B). However, fluorometric assays did reveal mean maximal fluorescence changes that were consistent with reductions in cell-surface expression of N753Q mutants (in RFU; WT: 1155; N747Q: 947; N753Q: 902; N747/753Q: 866). Meanwhile, the absence of a negatively-charged, cation-attracting glycan on hTRPA1 N747Q may provide a reason why maximal cell responses could be diminished despite slightly higher cell-surface expression.

### TRPA1 gating mechanics are altered by N747Q mutation

Fluorometric activity assays {*n*=4 independent experiments [Each fluorometric experiment (i.e., one independent experiment) described consisted of triplicate readings at each agonist concentration]} revealed that TRPA1 mutants lacking a glycosylation site at Asn^747^ exhibit decreased sensitivity to CA [EC_50_ (in μM), 95% confidence interval (CI)] (**WT**: 9.5, 8.7–10.4; **N747Q**: 32.5, 30.6–34.6; **N753Q**: 10.5, 9.3–11.9; **N747/753Q**: 20.9, 19.8–22.0; Figures 3B and 4). Although the EC_50_ of CA differed considerably between variants, differences in saturating concentrations were not obvious. Indeed, the steeper slope factors of the CA dose–response curves plotted for N747Q mutants relative to WT, combined with reduced RFU values at lower concentrations, might suggest that the absence of an N-glycan at Asn^747^ results in altered activation kinetics rather than reduced agonist affinity.

In theory, neither mutation should alter the affinity of lipophilic, sulfhydryl-/amine-reactive TRPA1 agonists, as they are known to interact with N-terminal cysteine and lysine side chains [26,38]. This gave rise to the idea that the functional differences between TRPA1 variants were the result of glycan participation in gating rather than agonist binding. To test this hypothesis, and to determine whether the altered sensitivity we observed was specific to N-terminal-domain-binding TRPA1 agonists, we performed additional fluorometric measurements applying a similarly-reactive, TRPA1-agonizing electrophile, AITC (*n*=2 independent experiments), as well as menthol (*n*=3 independent experiments), which activates TRPA1 via interaction sites along the S5 domain [19] (Figures 3C and 3D). The significant disparities between AITC and menthol dose–response curves were consistent with the variant-specific changes to CA sensitivity we observed [EC_50_ (in μM), 95% CI] (**AITC: WT**: 1.8, 1.2–2.5; **N747Q**: 4.2, 3.3–5.2; **N753Q**: 1.6, 1.1–2.3; **N747/753Q**: 3.0, 2.3–3.8; **menthol: WT**: 29.2, 24.8–34.3; **N747Q**: 68.9, 64.6–73.6; **N753Q**: 34.4, 30.1–39.2; **N747/753Q**: 58.0, 51.3–65.5; Figures 3C and 3D).

Finally, results from electrophysiological experiments also support the apparent functional significance of the N-glycan at Asn^747^. In whole-cell patch-clamp experiments, (*n*≥4 cells each) we observed similarly reduced sensitivity to lower concentrations of CA among hTRPA1 variants bearing the N747Q mutation [EC_50_ (in μM), 95% CI] (**WT**: 35.0, 22.2–55.0; **N747Q**: 141.0, 116.1–171.2; **N753Q**: 42.0, 28.5–62.0; **N747/753Q**: 142.3, 95.9–211.2; Figure 5A). Once again, these data support a 2- to 4-fold increase in agonist concentration required for half-maximal activation of hTRPA1 that is non-glycosylated at position Asn^747^.

### Variant-specific cooperative effects of temperature and CA

An apparent cooperativity between cold temperature and chemical agonists in the activation of TRP channels has been reported previously [19,40,41]. Bandell et al. [19] observed a roughly 9-fold lower EC_50_ of menthol on TRPA1 when experiments were conducted at an ambient temperature (23°C) rather than physiological temperature (37°C). Additionally, Pertusa et al. [12] identified an N-glycan at position Asn^934^ of TRPM8 that influenced channel temperature sensitivity. Accordingly, we ran fluorometric activity experiments at 23°C in parallel to those conducted at 37°C to determine whether such cooperativity between temperature and CA was evident and whether it could be influenced by N-glycosylation.

**Figure 6:**
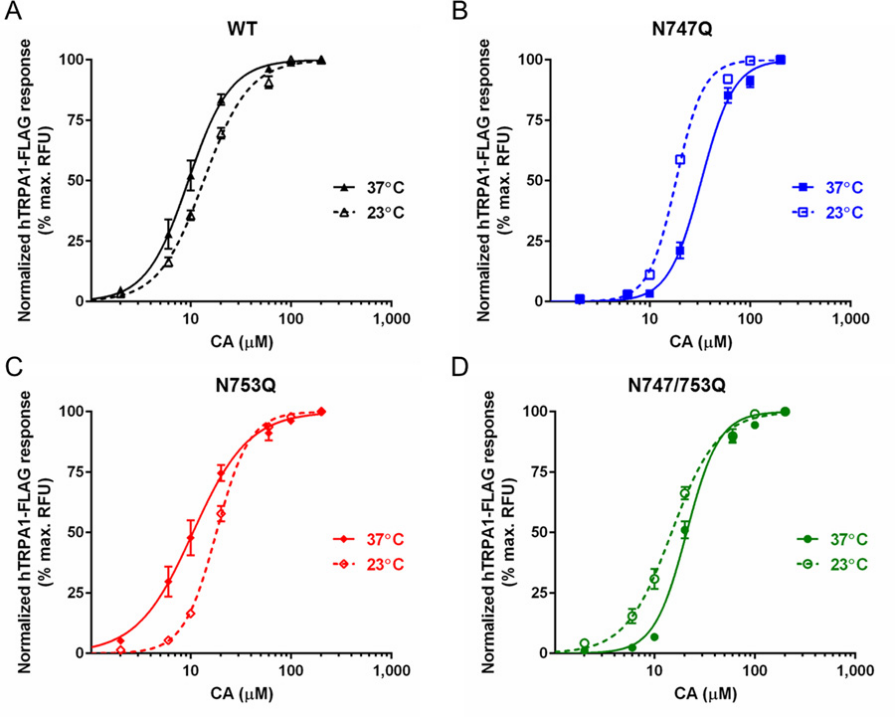
Cooperative effects of temperature and CA on TRPA1 activity Dose–response curves depicting normalized (% maximum RFU) membrane depolarization of HEK293 cells, at 23 and 37°C, expressing hTRPA1-FLAG glycoforms as measured on a FlexStation® 3 (*n*=4 independent experiments; data are expressed as mean ± S.E.M).

In our experiments (*n*=4 independent experiments), we observed a slight, albeit significant, decrease in the sensitivity of hTRPA1-FLAG WT and the N753Q mutant at 23°C relative to recordings at 37°C [EC_50_ (in μM), 95% CI] (**WT**: 13.5, 12.8–14.2; **N753Q**: 18.0, 17.3–18.7; Figures 6A and 6C). In contrast, the absence of the Asn^747^ glycosylation site led to enhanced sensitivity of TRPA1 at ambient temperature [EC_50_ (in μM), 95% CI] (**N747Q**: 18.2, 17.7–18.8; **N747/753Q**: 14.8, 13.7–16.9; Figures 6B and 6D). Despite these findings, we did not, however, observe any direct agonistic effect of noxious cold (as low as 4°C) on hTRPA1-FLAG expressed in HEK293 cells (results not shown). The cold-sensitivity of TRPA1 remains controversial, with our data supporting some findings [35,42,43] but contradicting others [27,44,45].

## DISCUSSION

Our study into the effects of N-glycosylation of hTRPA1 produced several new insights. In addition to confirming that both consensus sites can be modified with an N-glycan *in vitro*, we determined that the removal of these sites does not prevent functional assembly or cell-surface trafficking of the protein. On the contrary, it appears that the absence of the glycan at position Asn^747^ results in higher levels of protein localized to the cell surface. Additionally, we identified what appear to be important functional roles of N-glycosylation at the Asn^747^ consensus site. Specifically, we first observed that the N747Q mutation influences the efficiency of glycoprocessing of the N-glycan at Asn^753^. Later, we observed that the absence of the Asn^747^ glycan reduces hTRPA1 sensitivity to a variety of agonists, and those effects were more dramatic at physiological temperatures.

The effects of protein glycosylation are highly variable [18]. Because of this diversity, the task of characterizing N-glycans must–at least currently–be performed on a glycoprotein-by-glycoprotein basis. This appears particularly true in the case of TRP channels. For example–in evident contrast with hTRPA1–removal of the N-glycan on rat TRPV1 appears to enhance agonist sensitivity, with researchers observing a ∼5-fold reduction in the EC_50_ of capsaicin in non-glycosylated mutants [13]. As such, determining the specific function of N-glycosylation for each TRP channel plays a crucial role in expanding our understanding of both this protein family and, more broadly, the structural and functional protein traits that can be conferred by glycans. Several researchers have already contributed heavily in this regard [8–16], but many TRP channel-bound N-glycans remain unstudied.

Given the potential clinical significance of TRPA1 [46–53], insights into its structure and function could be of considerable value. Our data suggest a critical role of an N-glycan in TRPA1 gating mechanics, with its absence resulting in diminished channel sensitivity to lower concentrations of a broad array of agonists. Although that finding may be of greatest interest within the context of our research, it bears mentioning that confirming TRPA1 glycosylation and eliminating the possibility that it is required for either (a) protein assembly or (b) trafficking to the cell surface, is in itself highly relevant. This is especially true of the latter, as the N-glycosylation of TRP channels has been occasionally shown to have a profound impact on the levels of cell-surface expression [8,9,15].

Although the expression data we collected revealed that a greater proportion of hTRPA1 N747Q was localized to the cell surface than that of hTRPA1 WT, it is important to also consider our finding that the removal of the smaller glycan at position Asn^747^ resulted in more robust expression of mature (relative to immature) glycoprotein. This might suggest that the enhanced surface expression of this TRPA1 mutant arises due to its more efficient glycoprocessing rather than by the absence of the N-glycan at Asn^747^ *per se*. This would point to a critical role for the complex N-glycan at Asn^753^, namely that its presence promotes optimization of cell-surface expression. The expression patterns of hTRPA1 proteins bearing the N753Q mutation appear to support this idea. Both proteins possessing this mutation exhibited reduced total expression relative to WT and, consequently, reduced cell-surface expression. It is unclear whether these reductions signify less efficient protein processing or increased susceptibility to degradation. Regardless, it appears that optimal hTRPA1 expression patterns may rely on di-glycosylation.

The heightened level of mature hTRPA1 elicited by the N747Q mutation could arise from several possible causes. For example, it is becoming increasingly clear that sequences adjacent to the N-*X-*S/T consensus site can influence glycosylation and/or glycoprocessing [54–56]. Additional evidence highlights the influence that the distance between N-glycosylation acceptor sites can have on the efficiency of glycoprocessing [57]. Although one could hypothesize that an upstream N747Q mutation might result in unnatural glycoprocessing at position Asn^753^ (potentially resulting in the altered protein functionality), that contradicts our finding that the N747/753Q double-mutant produces similar agonist-induced behaviour to that of hTRPA1 N747Q. We find it more likely that close approximation of the glycosylation sites and/or the amino acids nearby the respective sequons are the cause of the apparent interplay between these two sites.

In light of the disparate levels of cell-surface expression we observed, it was surprising that the maximal TRPA1-specific responses observed in whole-cell patch-clamp and fluorometric assays did not differ significantly between TRPA1 variants. However, maximal functional responses may not directly correlate with TRPA1 cell-surface expression. Activation of TRPA1 in intracellular organelles, whose expression is not reflected in our membrane fraction WB analysis, may influence current and voltage-sensitive dye readouts. In addition, in the patch-clamp experiments, we may have had a bias towards selecting cells with a low level of TRPA1 expression, as these cells are likely to appear ‘healthier’ than cells with a higher expression level. Ultimately, the production of stable cell lines would be useful in resolving these discrepancies.

Given the consistency of our results from the functional analysis, both across different assays and between agonists with distinct reactivity profiles, we are confident in the functional significance of the glycan at position Asn^747^. As further support, the EC_50_ values we generated from fluorometric measurements of hTRPA1-FLAG WT-mediated membrane depolarization closely resemble those observed by other researchers using HEK293 cells in similar assays, for all three agonists [24,29,58], and we likewise observed IC_50_ values of the TRPA1-selective antagonist, HC-030031, that were consistent with the literature [51] (results not shown). These findings, to our thinking, validate the use of transiently-transfected HEK293 cells in fluorometric assays.

In our review of the literature, we did not uncover many full dose–response curves of TRPA1 generated in a whole-cell patch-clamp format in heterologous expression systems. However, Chung et al. [36] used precisely such a format to evaluate the response of TRPA1 to its agonist, eugenol. We found that TRPA1-expressing HEK293 cells were highly amenable to electrophysiological analysis, with serial dilutions of CA applied to single cells producing large, slowly-inactivating inward currents that waned only slowly upon removal of the agonist (Figure 4C). This enabled us to confirm the effects of the N747Q mutation, which once again demonstrated a notable loss of agonist-sensitivity. Although the EC_50_ values determined by whole-cell patch-clamp and fluorometric assays differed considerably, we considered this a consequence of the differences inherent to the respective assays. In addition to discrepancies in assay sensitivities, other factors, such as voltage control and/or the treatment of CA-naïve cells (in the FlexStation) compared with single-cells exposed to escalating CA concentrations (in patch-clamp experiments) likely contributed to the observed differences.

Recently, Deering-Rice et al. [59] published results from a functional analysis of non-glycosylated TRPA1 variants. Their work entailed analysing calcium influx after a single application of a saturating concentration of TRPA1 agonists to HEK293 cells transiently expressing either WT or N747A and/or N753A TRPA1 variants. To rule out disparate sub-cellular protein distribution as a cause of disparate channel responses, the researchers endeavoured to activate intracellular TRPA1 in isolation by applying TRPA1 agonists in the presence of the membrane-impermeable TRPA1 inhibitor, ruthenium red. Although they found diminished responses among TRPA1 variants bearing the N753A mutation, they did not–as we did–identify significant differences in protein distribution. We believe this is probably a consequence of the different analytical approaches, namely the use of immunoblotting of isolated cell-surface proteins compared with an intracellular activity assay, to determine differences in TRPA1 cell-surface expression.

The influence of N-glycosylation on the apparent cooperative effects of temperature and CA was a surprising finding in the present study. In particular, it is interesting that the presence of both glycosylation sites appears to optimize TRPA1 agonist-sensitivity at physiological and ambient temperatures. Perhaps in the case of TRPA1, di-glycosylation served as a kind of evolutionary calibration, especially in light of the fact that the Asn^753^ glycosylation site is more evolutionarily recent, with cold-blooded amphibians and reptiles lacking it. It would be interesting to see whether similar patterns are present among other thermosensitive TRP channels.

There are some limitations inherent to this analysis that bear acknowledgement. Specifically, it remains unclear whether TRPA1 N-glycosylation occurs in native cell lines *in vivo*. However, results published by other research groups consistently indicate that TRP channels are N-glycosylated *in vivo* [12,14], supporting extrapolation of findings obtained *in vitro*. In addition, we cannot exclude that the Asn-Gln mutations induce confounding changes to protein structure that may obscure other effects of glycosylation. Also, there were multiple TRPA1 subunit glycoforms present in single cells, as evidenced by the doublet bands observed in single samples in WB analysis. Individual TRPA1 variant glycoforms may contribute disparately to functional readouts, and the experiments we performed effectively aggregate these contributions.

Finally, TRPA1 is potentiated and inactivated by Ca^2+^ [60]. It is possible that WT and mutated TRPA1 channels analysed in our experiments differ in their permeability to Ca^2+^, which in turn could affect current amplitudes through Ca^2+^-dependent TRPA1 potentiation [60]. One might consider such disparities as an alternative explanation for the observed discrepancies between our expression and functional data. However, it is unclear what effects such disparities would have, if any, on the dose–response relationships between agonists and the TRPA1 variants under the experimental design we applied. In our experiments, the absence of significant differences in maximal TRPA1-specific response supports the statistical comparison of normalized dose–response curves. Furthermore, we did not observe a discernible pattern in TRPA1 channel function that was suggestive of the presence of confounding structural changes or disparate Ca^2+^-dependent potentiation brought on by the mutation to either site. On the contrary, the two variants bearing the N747Q mutation exhibit very different expression patterns but produce similar maximal signals and dose–response curves when stimulated by diverse agonists and under different experimental approaches.

One of the common features of TRP channels is their functional and structural versatility. They are typically polymodally activated, ubiquitously expressed, and can both hetero- and homo-multimerize [1–7]. The revelation that TRPA1 behaviour can be modified by altered glycosylation patterns potentially unlocks even more functional diversity. Glycosyltransferases are differentially expressed during development and across human cell-types [61–63]. Therefore, there is some potential that cell-type-specific transferase expression could ensure the targeted presence of functionally unique TRP channels, allowing proteins with identical primary structures to accommodate different functional roles. In the context of thermosensitive TRP channels, processes such as glycosylation may also be a means to optimize function over the range of temperatures to which an expression locus is typically exposed. For example, sensory receptors innervating the skin are exposed to lower temperatures than that of the core, and the functional demands of these respective environments could likewise differ.

The present study provides new insights into the complex channel behaviour and its tightly controlled regulation. Our confirmation of TRPA1 di-glycosylation, and the indications that this modification carries considerable functional importance for the protein, enhances our understanding of not only TRPA1 behaviour, but also of the broader TRP channel family as well as of the versatility of N-glycans.

## Abbreviations

AITC: allyl isothiocyanate
CA: cinnamaldehyde
CI: confidence interval
CL: (whole)-cell lysate
ConA: concavalin A
CS: cell-surface
GAPDH: glyceraldehyde-3-phosphate dehydrogenase
ORF: open reading frame
RFU: relative fluorescence units
RT: room temperature
TRP: transient receptor potential
TRPA1: TRP channel ‘ankyrin’ 1
WT: wild-type
